# *Facilitators* and Barriers to Nurse Practitioners Prescribing Methadone for Opioid Use Disorder in Nova Scotia: A Qualitative Study

**DOI:** 10.1177/0844562121996222

**Published:** 2021-02-20

**Authors:** Aaron E. Bates, Ruth Martin-Misener

**Affiliations:** 1 Faculty of Medicine, Dalhousie University, Halifax, Nova Scotia, Canada; 2 School of Nursing, Dalhousie University, Halifax, Nova Scotia, Canada

**Keywords:** Nurse practitioners, methadone, opioid use disorder, addiction, prescription, primary care

## Abstract

**Background:**

Opioid use has escalated dramatically resulting in an increase in deaths. Access to treatment for opioid use disorder (OUD) is poor. The addition of nurse practitioners (NPs) as prescribers of methadone for OUD offers potential for improving access. Little is known about what support NPs will require as they prescribe methadone.

**Purpose:**

This paper identifies facilitators and barriers to NPs prescribing methadone.

**Methods:**

In this qualitative study, in-person and phone semi-structured interviews were conducted with 18 participants. Participants included NPs (n=5), physicians (n=5), and stakeholders including members of professional regulatory bodies and government, academics and other clinicians (n=8). Interviews were recorded, transcribed, and analyzed using thematic analysis and software (NVivo 12.4.0) for data management.

**Results:**

Four themes emerged: 1) Pervasive Barrier of Stigma; 2) Perceived Complexity of Patients Living with OUD; 3) NP Education and Practice Supports and; 4) Health Care Context and NP Role Implementation.

**Conclusions:**

Barriers and facilitators to NP prescribing are similar to those encountered by physicians. Factors unique to NPs include the identification of role clarity as a facilitator and navigation of physician networks as a barrier. Research conducted with current NP methadone prescribers is required to evaluate implementation of this service.

## Background and purpose

Worldwide the number of opioid users has increased dramatically in recent years to an estimated 35 million people (United Nations Office on Drugs and Crime, 2019). Blanco and Volkow (2019, p.1760) define opioid use disorder (OUD) as “a pattern of opioid use associated with a range of physical, mental, social, and legal problems, and with increased mortality leading to clinically significant impairment or distress.” North America sees a high number of deaths from synthetic opioid overdose (Global Burden of Disease 2016 Alcohol and Drug Use Collaborators, 2018). In Canada there were 3,799 apparent opioid related deaths in 2019. This compares to 4,372 deaths in 2018 and 4,150 deaths in 2017 (Government of Canada, 2020b). In 2019, Nova Scotia’s number of opioid related deaths (57), and its rate of deaths (5.9 per 100,000 population) is significantly less than British Columbia (1,002 deaths, 19.8 deaths per 100,000 population) and Ontario (1,509 deaths, 10.4 deaths per 100,000 population) yet greater than figures for neighbouring New Brunswick (29 deaths, 3.7 deaths per 100,000 population) (Government of Canada, 2020b). The national rate of opioid related deaths in Canada in 2019 was 10.1 per 100,000 population (Government of Canada, 2020b). The decrease in deaths in 2019 is likely to be reversed in 2020, due to changes in the patterns of substance use associated with the COVID-19 pandemic (Government of Canada, 2020a). In two of three scenarios projected by the Public Health Agency of Canada, deaths due to opioid use in 2020 will surpass the previous peak, in late 2018 (Government of Canada, 2020a). Preliminary data indicate that the number of deaths is increasing, with First Nations people disproportionately affected (Kapelos, 2020; Tasker, 2020).

Medications, including methadone and buprenorphine-naloxone, are a standard of care for treatment of OUD (Bruneau et al., 2018; Dowell et al., 2016; Volkow et al., 2019) and prescribers who treat OUD, which in Canada includes nurse practitioners (NPs) and physicians, should be familiar with both. While legislative barriers preventing NPs from prescribing methadone were recently removed, organizational and other structural barriers may remain. Little is known about what support NPs will require as they take on this responsibility. To address this gap in knowledge, this study explored the facilitators and barriers to NPs prescribing methadone and how facilitators may be enhanced, and barriers mitigated.

OUD is compounded by concurrent mental health conditions (Harris et al., 2019). Among adults in the United States with OUD, 64% were found to have a diagnosed mental illness in the past year (Jones & McCance-Katz, 2019). Nearly 27% of adults with OUD had a mental illness of sufficient severity (including mood and anxiety disorders) that function was impaired (Jones & McCance-Katz, 2019). Alcohol and cannabis use disorders are associated with opioid misuse, particularly in the context of chronic pain (Rogers et al., 2019). In addition to the risk of unintentional overdose, OUD is associated with other risks including injection-related infections such as HIV, hepatitis and sepsis (Dooley et al., 2012; Global Burden of Disease 2016 Alcohol and Drug Use Collaborators, 2018) and suicide (Bohnert & Ilgen, 2019).

Methadone is an opioid agonist and binds to opioid receptors in the central nervous system (Lexicomp, Inc, 2020b). Buprenorphine is an opioid agonist with high affinity to mu receptors in the central nervous system (Lexicomp, Inc, 2020a). The naloxone component in buprenorphine-naloxone deters misuse of the drug, as intravenous or intranasal administration of buprenorphine-naloxone may cause rapid withdrawal of buprenorphine (Canadian Pharmacists Association, 2019). Both treatments are intended to control the withdrawal symptoms of opioids while attenuating cravings (Bruneau et al., 2018). Recent Canadian guidelines recommend buprenorphine-naloxone as the first-line and preferred initial treatment (Bruneau et al., 2018). While both medications are equally efficacious, buprenorphine-naloxone is a safer option than methadone because of a lower risk of respiratory depression (Bruneau et al., 2018). Methadone may be more effective than buprenorphine-naloxone in controlling withdrawal symptoms in patients with higher tolerance and frequent opioid use and is potentially the preferred option for this patient group (Bruneau et al., 2018). Nurse practitioners (NPs) prescribe buprenorphine-naloxone in Nova Scotia (Nova Scotia College of Pharmacists, 2017).

The practice of NPs in Nova Scotia is governed by the Registered Nurses Act. The Act specifies that the NP may, among other privileges, “order and interpret screening and diagnostic tests, and recommend, prescribe or reorder drugs, blood, blood products and related paraphernalia” (Province of Nova Scotia, 2018, section ak). With the approval of the New Classes of Practitioners Regulations (NCPR) under Canada’s Controlled Drug and Substances Act (CDSA) in 2012, the federal barrier to NP prescribing of controlled substances, with some exceptions, was removed. This facilitated provincial and territorial legislative and or regulatory changes. In 2014 the Government of Nova Scotia added the College of Registered Nurses of Nova Scotia (CRNNS) as a licensing authority under the *Prescription Drugs Monitoring Act*, allowing NPs to prescribe controlled substances (Health and Wellness, 2014). These prescribing rights included OUD treatment medications, with the exception of methadone. In the March 7, 2018 issue of the Canada Gazette (Part II: Volume 152, Number 6) (Government of Canada, 2018), it was announced that the requirement for a Health Canada exemption for methadone prescribers would be lifted and NPs were included as methadone prescribers. The changes came into effect in May 2018. The Nova Scotia College of Nursing (NSCN, formerly CRNNS) expects that NPs will prescribe methadone based on current evidence-informed guidelines, best practice and employer policy and that they will maintain their competence through education and practice (Nova Scotia College of Nursing, 2019a). Similarly, the Nova Scotia College of Physicians and Surgeons continues to expect that methadone prescribers “will do so only with the appropriate knowledge and training” (College of Physicians & Surgeons of Nova Scotia, 2018a, Para. 4).

In Nova Scotia, methadone has historically been the first-line treatment for OUD (Dooley et al., 2012). Prior to removal of the exemption requirement, Nova Scotia had approximately 80 physician methadone prescribers (College of Physicians & Surgeons of Nova Scotia, 2018b; Laroche, 2017). Research regarding barriers to physician prescribing revealed that a perceived lack of support, a lack of experience and the perception that methadone patients are “difficult” were the primary impediments (Dooley et al., 2012, p. 6).

It is not yet known whether the removal of the exemption requirement for all methadone prescribers or the addition of NPs as new methadone prescribers will increase access to methadone treatment for patients with OUD. NPs are expected to have “a leadership and advocacy role in the healthcare system” (Nova Scotia College of Nursing, 2019b, p. 7) and to “develop, implement and evaluate initiatives to promote health and to prevent injury and illness” (Nova Scotia College of Nursing, 2019b, p. 7). In this context, NPs have an important role in addressing the public health challenge of improving access to and quality of treatment services for OUD.

## Methods and procedures

This study used a qualitative descriptive design (Sandelowski, 2010). A qualitative descriptive design was selected because little is known about the phenomena of NP prescribing of methadone, thus justifying a method that comprehensively summarizes participants’ perspectives while staying close to their own words. Both authors of the paper are or were NPs and have experience with NP and physician networks in the province. This enabled recruitment and development of trust with interview participants. Participants were provided the opportunity to describe events and circumstances as they understood them in semi structured interviews.

### Sample

The final sample included 18 participants, five of whom were NPs, five were physicians and eight were stakeholders. To protect the confidentiality of participants limited demographic information is provided. Participant NPs (n=5) worked in primary care. None of the NP participants were methadone prescribers. At the time of the research (summer 2018) there were no NP prescribers of methadone in Nova Scotia. These NPs had either an interest in addictions or health policy. All NP participants were female, and the length of their NP practice experience ranged from fewer than 5 years to more than 20 years. Physician participants (n=5) were current or past methadone prescribers and most had experience working with NPs. Participants were both male and female, worked in both urban and rural settings and worked in both family and specialist practices (pertaining to mental health and addictions). Stakeholder participants (n=8) were both male and female clinical and non-clinical persons engaged with the treatment of OUD including decision makers in professional regulatory bodies, health organizations and government, university professors and other clinicians. Since Nova Scotia is a small province, to protect the identity of these participants we have only used the term “stakeholder” when attributing their quotes.

### Recruitment

Participants were recruited purposively and with a snowballing method from each of Nova Scotia Health’s (NSH’s) four management zones. Initial participants were identified using the professional networks of the authors. Participants then identified further potential participants from among their networks. This technique allowed the uncovering of knowledge embedded in organic social networks (Noy, 2008). A risk with using a snowball approach and social networking for recruitment is selection bias. However, in this study it was necessary given the newness of NPs’ prescriptive authority and lack of a registry with the names of all NPs who were methadone prescribers.

### Data collection and analysis

In person and telephone semi-structured interviews were conducted by the first author who is a former NP and is now a medical student at Dalhousie University. An abbreviated interview guide is included (see Appendix). Interviews were structured around scripted questions with follow-up prompts developed by the first and second author, who has significant experience in qualitative research.

In person interviews took place in the participant’s workplace or another location. Interviews were audio recorded and lasted from 30 to 60 minutes. Rigor was demonstrated through four criteria associated with the trustworthiness of qualitative descriptive research (Bradshaw et al., 2017). Credibility occurred through development of a trusting relationship during the interviews in which participants felt free to speak, as well as by ensuring accurate transcription (Bradshaw et al., 2017; Milne & Oberle, 2005). Confirmability and dependability were achieved through an audit trail and frequent meetings of the research team for critical review of coding and to discuss emerging findings (Bradshaw et al., 2017; Milne & Oberle, 2005). Transferability was demonstrated through purposive sampling and providing rich description that was attentive to context (Bradshaw et al., 2017; Milne & Oberle, 2005). To ensure rigor, both authors reviewed the same two transcripts and independently identified initial codes. These codes were discussed, and a final coding system developed.

All participants provided written consent. The study protocol was approved by the Nova Scotia Health Authority Research Ethics Board (NSHA REB ROMEO File #: 1023362). Interview transcripts were read and reread for verification and entered into qualitative analysis software for data management (NVivo 12.4.0).

Transcripts were coded using the coding system and employing Glaser’s constant comparison methods within interviews and sequentially (Glaser & Strauss, 1967). Discrepancies in coding were resolved through discussion and consensus. Following coding, themes were identified through discussion between both authors who met biweekly during the analysis phase. These themes when interpreted described the facilitators and barriers to the prescription of methadone by NPs. Similarities and differences in the perspectives of NP, physician and stakeholder participants were examined.

## Results

Four themes were identified that influence the NP prescription of methadone: 1) The Pervasive Barrier of Stigma; 2) The Perceived Complexity of Patients Living with OUD; 3) NP Education and Practice Supports; and 4) Health Care Context and NP Role Implementation. Participants are identified by pseudonyms.

### Theme 1: The pervasive barrier of stigma

Stigma permeated much of the concern that participants expressed regarding patients experiencing OUD. Three subthemes were identified: personal beliefs, health care provider and staff beliefs; and public stigma.

#### Personal beliefs

For some participants, stigma presented as personally held beliefs rooted in societal perceptions. Anna, NP, identified that some NPs view patients with OUD as less deserving of care than other patients, *“a lot of people’s perceptions around addictions is ‘Oh they got themselves into this they can get themselves out, why are we wasting tax payer dollars on this sort of foolishness?”* One NP acknowledged how common stigma was, and that she was not immune, “*There’s always the stigma right? You’re even probably hearing some stigma from me*” (Francesca). Many participants across all groups acknowledged that some prescribers may choose not to include methadone as a part of their practice for personal reasons. Francesca, NP, said, “*There could be certain people because of religious reasons or who knows what … Some people are just like ‘no, that’s still drugs and I’m not going to be a part of that*.’”

#### Health care provider and staff beliefs

NPs and MDs concurred that resistance, by colleagues and other office staff, to offering methadone treatment deters NPs from seeking this area of practice. As clinicians expressed that they depend heavily on clinical colleagues and on administrative staff, there was little motivation for NPs to attempt to integrate methadone treatment into practices. In some workplaces, methadone was explicitly excluded as a treatment modality.

*But in some sites … it was a clinic decision they would not, full stop, prescribe methadone … [a job interview] questions, was, “Will you be ok with not providing that service, because it’s not one that we’re interested in entertaining” (Tessa, NP).*

Francesca also raised stigma as a potential issue with office staff. “*Yeah, could be other staff … someone else goes ‘no way, I don’t want to be a part of this [prescribing methadone], I’m not comfortable with this, we’re already overwhelmed*.’” Another NP felt that some NPs would be uncomfortable with methadone treatment as “*Methadone still gets you high, to certain extent, it is still a pretty potent agonist*” (Cecilia, NP).

Craig [physician], acknowledged that stigma from physician colleagues may be a barrier for NPs interested in prescribing methadone. “*If there’s a significant amount of discrimination or stigmatization amongst your colleagues, then that could create some issues.*” Stakeholders emphasized how stigma may be a barrier to NPs. “*Historically there’s a great deal of stigma attached to addictions clients. And so that may make the work look less appealing*” (Barbara, stakeholder). None of the stakeholders in our study, including academics, talked about experiencing or perpetuating stigma.

#### Public stigma

While it is encouraging that NPs acknowledged that stigma was often foundational to their reservations regarding the treatment of OUD, most NPs appeared resigned that stigma would remain a barrier:

*There’s stigma of just going everyday to the pharmacy and being there, exposed, people starring at you, you have to wait in line. I don’t know how to get around this … It’s all out in the open. I wish there was something we could do about that … It’s going to be difficult to be confidential and supportive and reduce stigma … I don’t know how that gets resolved* (Francesca, NP).

One NP reflected that stigma may be reduced through education and increased experience with this population, “*But again that might be where some more education and maybe hands on might come in*” (Evelyn, NP).

### Theme 2: Perceived complexity of patients living with OUD

All NPs, physicians and most stakeholders felt that the perceived complexity of the patient group would be a barrier to NPs prescribing methadone. Three subthemes were identified*:* multiple chronic health challenges; workload and resources; and risk of violence.

#### Multiple chronic health challenges

Bethany, a physician, explained the complexity of the concerns of methadone patients and pointed out the reluctance of providers to assume their care:

*The reason people get addictions is because a lot of things have typically happened to them. They’ve had huge trauma burdens. They have other medical complaints. They’re very complicated challenging patients. I don’t know if people [providers] want to take that on*.

#### Workload and resources

NPs suggested that, except for those internally motivated to work with this patient group, little incentive exists to begin working with a new, complex, patient group. NPs felt that they are able to have fulfilling primary care practices without the perceived complications of prescribing methadone. “*The current patient population and panel is already quite high, and so opening it up to more patients might be challenging”* (Tessa, NP).

Cecilia, NP, pointed out the challenges of the population and the lack of available community or hospital resources, *“If they’re self-medicating due to historical trauma or untreated psychiatric conditions … and they don’t have access to psychiatric care … what do you do?”* Cynthia, one of the stakeholders, further elaborated how perceived complexity of the needs of the patient group may act as a deterrent to NPs wanting to take on a methadone prescribing role:

*Physicians were provided with the [methadone] education … but then they didn’t always go on to provide the service … Part of it was the patient load and the amount of work that would be involved … that is when a collaborative team would help*.

Among physicians it was acknowledged that the time required to treat patients with OUD varied among practice settings. Bethany, physician, explained that she spent more time with patients as she addressed the social and psychological basis for addictions, “*I do intensive case management*.” In practice settings with multidisciplinary support:

*[The prescriber] … can see 40 people [a day], say follow-ups. Right. He writes the prescriptions. Based on the urine drug screen that’s done in the office for him, and the therapist who’s seen them, also in that building. Right … Pretty easy. Not hard (Bethany, physician).*

#### Risk of violence

Some NPs were concerned that prescribing methadone for OUD would expose them to a risk of violence, “*Many of us work in reasonably isolated conditions … and that’s frightening*” (Anna). Most physicians felt the risk of violence to be a perceived risk, rather than an actual risk:

*I think there is a perception that addicts are violent … and there’s no doubt that violence is a part of life of many addicts … I don’t think in my practice anyway, that there would be any significant difference between let’s say aggression from addicts as opposed to aggression from people with mental illness … I can certainly see that might be something that NPs might state as one of their concerns* (Craig, physician).

A physician in the sample agreed with Craig, “*Opioid people aren’t usually very violent. Those aren’t the ones you’re worried about … In fact, they’re all more likely to have a crime committed against them. So, I don’t think there’s any increase in fear of my personal safety*” (Bethany, physician).

### Theme 3: NP education and practice supports

Current NP education models were explored as a barrier. Some NPs saw a lack of access to ongoing education as a barrier. Two subthemes were identified: entry-level education and ongoing learning resources and supports

#### Entry-level education

Most participants felt that inadequate expertise in working with methadone patients was a barrier. All NP participants identified lack of knowledge as a barrier:

*So, you actually put people in the position as primary healthcare providers, of having to be one-stop shopping for multifaceted, complex issues that they don’t have the training to do, nobody is really up for that. Especially with a highly stigmatized population* (Cecilia, NP).

Participants felt that the structure of NP education discourages new NPs from choosing to prescribe methadone. NP informants revealed that they received little addictions training, and no methadone education, as a part of their initial education. “*We don’t do a lot of speciality training, we don’t have any specialty mental health or addictions training right now in the nurse practitioner program. And you really do want to have good skills for that kind of stuff*” (Evelyn, NP). When addictions are studied, their consideration is separate from other chronic health conditions. This separation identifies addictions and methadone as niche practice areas requiring specific and difficult to obtain knowledge.

Craig [physician], expressing a similar point of view, stated:

*There needs to be more [addictions education] while you’re getting your training … It [addictions treatment] becomes part of the curriculum like diabetes … that will do two things, it will define addiction as a chronic disease just like any other chronic disease … but it will also obviously provide a foundation of knowledge that will allow NPs to feel more comfortable.*

There was a belief among most NPs and physicians regarding the importance of an early introduction to opioid addiction and treatment in education programs:

*Exposing nurses, NPs, early on to substance use disorders, and effective management and the role that methadone plays in that — that’s essential for having people ready to recognize that this is part of their practice … it’s not an add on* (Evelyn, NP).

#### Ongoing learning resources and supports

Participants also spoke of the need for ongoing learning resources and supports in their practice. Some NPs felt confident about their ability to access supports while acknowledging that others may feel differently. “*I personally know of the resource that I can call and have called many times … Whether some others would feel, depending on the collaborative team they’re in, that they are in isolation*” (Tessa, NP). Tessa’s comfort in accessing support may be explained by the fact, in addition to her significant clinical experience, her NP role included efforts to connect other NPs to clinical practice resources.

Physicians tended to believe that appropriate support is available to NPs, “*there might be this perception of being isolated, but I think that it wouldn’t take much for any prescriber, any nurse practitioner, to be able to get the support they need if they try a little bit. I don’t see isolation as, the perception of isolation might be there, but I don’t see it as a real concern*” (Craig, physician). Some of the variance in the opinions regarding supports may be due to the fact that information is generally shared through informal physician networks. It is notable that physician participants generally believed that resources are accessible, while NP participants were ambivalent.

Most NPs and many physicians identified the potential for collaboration with other team members to be a strong facilitator to NP prescribing of methadone. “*The social worker met with the person, did some counselling, made sure everything was OK … with regards to social determinants of health. That’s the ideal setup*” (Francesca, NP).

Craig, a physician, felt that the potential for collaboration was present for NPs who seek it: “*There is reasonable access electronically and over the phone … I think that it wouldn’t take much for any prescriber, any nurse practitioner, to be able to get the support they need.*” Most stakeholders viewed collaboration as critical for methadone prescription. Daniel said, “*From a quality perspective I would be very leery of anyone, physician/NP in solo practice … the best evidence for treating addiction is that it’s an interdisciplinary approach*.” Methadone treatment programs in Atlantic Canada have historically followed this biopsychosocial model, combining pharmacological treatment with services addressing the social and psychological dimensions of addiction (MacNeill et al., 2020).

Participants identified the consequences of a lack of support. Several physicians spoke about personal and professional burnout as a barrier to adding methadone to their practices. Bethany stated, “*Maybe people don’t want [to add] another chronic disease that’s really, really hard [to patient rosters that are already full and complex]. Maybe people feel helpless … So then they’ll put up the walls*” (physician). NPs had varying opinions regarding the availability and accessibility of methadone support systems. Some NPs felt that institutional supports weren’t available, “*so if I’m willing to prescribe methadone that’s great, but … we have to have nursing involvement, we have to have clerical involvement, et cetera and the mental health availability is not there*” (Anna, NP). Another NP appeared unsure about access to methadone education, “*I think … that email went out about the education … I think that’s probably a place to start … and maybe like support from your peers and collaborating physicians in the area*” (Evelyn, NP).

### Theme 4: Health care context and NP role implementation

Two sub-themes were identified: regulatory challenges and acceptance of the NP role. Regulatory challenges were identified as a barrier. Acceptance of the NP role was uncovered as a facilitator.

#### Regulatory challenges

All NPs, and nearly all other participants, were aware of NP methadone prescribing. One stakeholder believed that the general confusion about methadone prescribing since Health Canada’s changes may be a barrier for NP prescribing. “*The restrictions regarding having the methadone specific license, those have evaporated leaving behind a bit of a questionable wasteland of people trying to figure out what they’re doing*” (Peter).

Historically, NPs wishing to make methadone prescription a part of their practice have encountered difficulties. Communication with regulatory and government agencies regarding scope of practice and training resources was historically difficult. Anna, an NP, explained that prior to the removal of the methadone exemption she took methadone prescription courses “*because I was approached by Mental Health Addiction Services to begin prescribing … and although I took the courses there was no ability to gain an exception from the College of Nursing*.” Tessa, an NP, expressed a similar sentiment, “*the educational module and preceptorship and the work you need to go through in order to enact the authorization, it’s not something most are interested in doing*.” Anna, although initially motivated to treat patients with OUD, has not (at the time of this research) included OUD treatment in her practice. She felt that institutional supports were insufficient and that, “*I think it’s gonna take a few brave souls to get out there and actually walk the walk a little bit before you’ll see big numbers of NPs willing to do this*” (Anna, NP).

NPs were unanimous that compensation was not a barrier. As Cecilia explained, “*It’s a non-factor. So, NPs are reimbursed by salary … I see zero barriers in terms of reimbursement*.” Other participants were unclear whether compensation would be a barrier. “*Nurse practitioners who are kind of on a salary, there’d be no financial incentive for them to participate … So I’m not sure if that serves as a barrier or not*” (Tom, stakeholder).

#### Acceptance of the NP role

Some physicians stated that a lack of understanding by physicians of the NP role was a barrier:

*Barriers, the role of NPs from a physician perspective … I think most physicians are somewhat unsure of what the role of NPs is or should be. And, I think that’s a bigger question about the relationship between NPs and physicians and the role of NPs in the health system … Sometimes they are connected with physicians, sometimes they aren’t. That’s a bit confusing. I think some physicians feel threated by NPs”* (Andrew).

One physician expressed that NP prescription of methadone for OUD was not a good idea, saying, “*some of the things I’ve seen from NPs, because they don’t have the medical training, can be concerning too. And these are complex patients”* (Bethany).

Other participants were optimistic that NPs would be accepted both by physician colleagues and by the public. “*Once physicians, who may be opposed to the NP, work with the NP, they understand what value is added*” (Daniel, stakeholder). They identified relationships among team members as complex, particularly when changes such as OUD prescription by NPs are introduced. Alexa, stakeholder, elaborated:

*Every time you create new roles and responsibilities for any member of the interprofessional team … there’s care and attention that needs to be paid to the team as these new functions are being assumed … particularly among nurses and physicians or NPs and physicians.*

One participant identified that a way to mitigate tension associated with roles and responsibilities of team members is through the involvement of regulatory bodies. “*I think some form of publication going around to physicians as it rolls out is key, highlighting the fact that NPs are going to be a very important part of this care plan or handling this crisis … your College supports it*” (Peter, stakeholder).

## Discussion

Worldwide access to treatment for concurrent mental health and OUD is poor (Harris et al., 2019). The addition of NPs as prescribers of methadone for OUD offers potential for improving access. This study is unique in that it captured the perspectives of NPs, clinical and policy stakeholders as well as physicians about NP prescribing of methadone. Numerous barriers to NPs prescribing methadone for OUD were identified. These included: stigma; limited NP education regarding addictions and methadone; the design of NP curricula (with addictions studied separately from other chronic conditions); unclear communication from regulatory agencies; a lack of institutional support; and the perceived complexity of patients with OUD. Fear of violence was a barrier for some NPs. Facilitators included access to collaborative practices and physician clarity of the NP role.

While numerous studies have looked at the role of NPs in the treatment of OUD, most of this research was conducted in the United States and focused on NP prescription of buprenorphine (Burda-Cohee, 2006; Fornili & Burda, 2009; Fornili & Fogger, 2017; O'Connor, 2011). One American study regarding NP prescription of buprenorphine identified that NPs operating in environments with fewer restrictions on practice were more likely to prescribe buprenorphine for OUD (Spetz et al., 2019). A second American study (Moore, 2018) investigating the prescription of buprenorphine-naloxone identified facilitators and barriers similar to the findings of this study: stigma and difficulty accessing supportive colleagues were significant barriers, while internal motivation was a significant facilitator (Moore, 2018). The studies that have investigated barriers to prescribing of methadone for OUD have focussed on physicians (Chan et al., 2014; Dooley et al., 2012; Fraeyman, et al., 2016; Livingston et al., 2018). A recent Canadian study that examined client experience in three different methadone treatment programs (comprehensive programs, low-threshold/high-tolerance (LTHT) programs, and fee-for-service (FFS) programs) in one Atlantic Canadian city identified a role for NPs in the delivery of methadone (MacNeill et al., 2020). Interestingly no one in our study discussed NP prescribing in relation to models of treatment. This may be because we did not specifically ask this question, or it may be that this question has not yet been considered from a policy perspective. Although the MacNeil et al. study did not investigate facilitators or barriers to NP prescribing of methadone, it identified the importance methadone clients give to counselling and other supports. This aligns with the perceptions of participants in our study who acknowledged this need and were hesitant about their current capacity to provide this service.

Our study determined that some barriers to NP prescription of methadone are similar to barriers experienced by physicians. The literature states that physicians feel uncomfortable treating this patient group (Chan et al., 2014; Dooley et al., 2012; Fraeyman et al., 2016; Livingston et al., 2018). Physicians reported that patients with OUD were difficult to manage and that they (physicians) lacked training and support (Chan et al., 2014; Dooley et al., 2012; Fraeyman et al., 2016; Livingston et al., 2018). Our study identified similar barriers for NPs. The literature supports this finding, and describes efforts to increase NP training in addictions (Creamer & Austin, 2017).

The importance of early exposure to addictions and addictions treatment in health professionals’ education was stressed across all groups of informants, reinforcing what is found in the literature. Chan et al. (2014) identified that physicians exposed to addictions during their training are nearly twice as likely to provide methadone treatment for OUD. Little research has been done about NP or nursing students’ perceptions about substance use disorder. One study conducted in the United States assessing the knowledge and perceptions of first year nursing students towards people with OUD found stigma and bias improved following an educational intervention (Lanzillotta-Rangeley et al., 2020). Another study conducted in a different university and state with undergraduate and NP students found knowledge and attitudes improved with education (Williams et al., 2020). Since the studies were not longitudinal, it is not known whether the change persisted over time. Although no Canadian studies focussed on education were found, we noted that the federal government recently invested significant funds to the Canadian Association of Schools of Nursing to update entry-level educational materials about substance use and the opioid crisis for nursing, pharmacy, and social work programs (Health Canada, 2019).

Participants endorsed the importance of methadone prescribers having access to ongoing methadone expertise. This is reinforced in the literature (Dooley et al., 2012; Livingston et al., 2018). A novel finding of this study is that while physician participants indicated existing OUD-focussed collaborative networks as open to the participation of NPs, NP participants were ambivalent. A possible explanation is that information about methadone resources has traditionally been transmitted via physician networks thus limiting NP access. NP ambivalence to participating in physician networks may also be rooted in historical challenges with interprofessional collaboration and resistance from organized medicine to the NP role (Donald et al., 2010; Martin-Misener & Bryant Lukosius, 2016). Similarly, the new role for NPs in Medical Assistance in Dying (MAiD) has also created ambivalence for some NPs (Pesut, Thorne, Schiller, Greig, & Roussel, 2020; Pesut, Thorne, Schiller, Greig, Roussel, et al., 2020).

A finding of this study is that NPs viewed the threat of violence as a barrier while physicians did not. Workplace violence against Canadian nurses is common in hospital and community settings (Havaei et al., 2020; Registered Nurses Association of Ontario, 2019). In a study of workplace violence, Havaei et al. (2020) identified that the majority of British Columbia nurses had experienced physical assault (86.4%), threat of assault (91%), emotional abuse (89.4%) and verbal sexual harassment (70.8%), while a significant minority had experienced sexual assault (20.0%) (p. 6). More than 90% of respondents in the study identified as female. This is consistent with other literature that identifies that workplace violence is gendered with more women than men affected (Lanthier et al., 2018; Lemelin et al., 2009). This awareness may have influenced how NPs in our study responded particularly since all were female. Only one of the five physicians in our study was female and she did not identify the threat of violence as a barrier. These differences may be due to broader cultural and gendered perceptions and or experiences of violence. A recent study from the Unites States found that over 50% of physicians and NPs working in pain management clinics had experienced violence in their workplace (Moman et al., 2020).

A further novel finding is that the lack of NP role clarity was identified as a barrier, while NP role clarity was identified as a facilitator. Encouragingly, it was also found that physician familiarity with NPs tended to increase physician comfort with the NP role in methadone prescribing. The study also revealed that exposure of physicians to the NP role does not universally lead to increased comfort. Some physicians remained skeptical despite exposure to NPs. Our findings are consistent with other studies. The literature examining role clarity and its impact on physician acceptance of the NP role crosses many settings including primary care (Brault et al., 2014; Donald et al., 2010), long-term care (Kaasalainen et al., 2010) and acute care settings (Donelan et al., 2020; van Soeren & Micevski, 2001).

Finally, participants in our study identified that OUD and its treatment with methadone is stigmatized. As a recent Canadian study found, stigmatization is an issue that has consequences for people who are undergoing methadone treatment. When patients are the recipients of traumatizing stigmatizing comments and behaviors from health care providers it can impact their comfort and willingness to access treatment (Woo et al., 2017). An American survey found that stigma among the public was associated with support for punitive health policies (Kennedy-Hendricks et al., 2017). As Kameg (2019) has noted, some of the stigma associated with OUD is directly related to, and fueled by, the language used to describe OUD and its treatment. Allen et al. (2019) reinforce that stigmatizing language propagated by providers and the public limits access to OUD treatment. The ability of NPs to offer this important therapy is an opportunity to provide care to an underserved population.

Our data collection occurred before any NPs in Nova Scotia had started to prescribe methadone, nor had there been health care system planning for how this new role for NPs would be integrated into existing structures and systems. Simply changing legislation will not result in development of a model of care that will meet OUD patients’ needs. The findings from our study indicate that NP implementation of methadone prescribing is complex and there is a need for systematic planning and evaluation as well as attention to NP education needs. Structures in NP education that separate addictions from other chronic conditions may inadvertently contribute to stigmatization. Future research should examine knowledge and attitudes of Canadian NP students and NPs in practice to explore the nature of stigma. Ultimately research is needed to understand both patients’ experience with NPs as providers and system level outcomes with NPs as methadone providers.

## Limitations

The regulatory guideline published by the NSCN announcing that NPs were able to prescribe methadone was initially published by the CRNNS in May 2018 (Nova Scotia College of Nursing, 2019a). The interviews for this study were conducted in July and August 2018. At this time, there were not any NP methadone prescribers in Nova Scotia. The responses provided by NPs in this study reflect what NPs anticipated would be facilitators and barriers to methadone prescription. It would be worthwhile to repeat this study, and to include NPs that are methadone prescribers within the sample. Since the initial participants were identified using the professional networks of the authors and then using a snowball approach, some selection bias is possible. The rigor of the study was ensured by maintenance of an audit trail, frequent meetings of the investigators to discuss methods and providing rich description of findings.

This research considered primarily the policy and legislative context of Nova Scotia. Although the scope of practice of NPs is similar across Canadian jurisdictions, the transferability of the findings of this study may be influenced by local contexts. Further research with a national focus is necessary to accurately determine facilitators and barriers to NPs prescription of methadone in other parts of Canada.

## Conclusion

Barriers and facilitators to NP prescribing are similar to those encountered by physicians. Factors unique to NPs include the identification of role clarity as a facilitator and navigation of physician networks as a barrier. Successful implementation of NP prescribing of methadone requires changes to current models of OUD care. The central role that stigma plays in limiting access to OUD treatment must be addressed beginning with NP education programs. Research conducted with current NP methadone prescribers is required to evaluate implementation of this service.

## Appendix

*Below is an abbreviated version of the Interview Guide.*

1. Tell me about your current and past involvement in planning and or delivering health or social care for people with addictions, and in particular, with methadone services.
2. What services are currently available for people addicted to opioid (prompt-narcotic) drugs in the community or communities in which you work?
3. Tell me what you know about who is able to prescribe methadone for opioid use disorder in Nova Scotia.
4. A Health Canada exemption is no longer required to prescribe methadone. In Nova Scotia Nurse Practitioners may now prescribe methadone. Do you think many NPs will chose to do this?
5. If you *do* think that many NPs will prescribe methadone, what factors will encourage them to do so?
6. If you *do not* think that many NPs will prescribe methadone, what factors do you think will prevent them from doing so?
7. What are your suggestions for improving prescribing of methadone by NPs in Nova Scotia?
8. Is there anything else you think is important to developing an understanding of the facilitators and barriers to NP prescribing of methadone that you would like to share?
9. Is there anyone that you suggest I speak with, to further our understanding of the facilitators and barriers to NP prescribing of methadone?
